# Doped or Quantum-Dot Layers as In Situ Etch-Stop Indicators for III/V Semiconductor Reactive Ion Etching (RIE) Using Reflectance Anisotropy Spectroscopy (RAS)

**DOI:** 10.3390/mi12050502

**Published:** 2021-04-29

**Authors:** Guilherme Sombrio, Emerson Oliveira, Johannes Strassner, Johannes Richter, Christoph Doering, Henning Fouckhardt

**Affiliations:** Research Group Integrated Optoelectronics and Microoptics (IOE), Physics Department, Technische Universität Kaiserslautern (TUK), P.O. Box 3049, D-67653 Kaiserslautern, Germany; candido@rhrk.uni-kl.de (E.O.); strassner@physik.uni-kl.de (J.S.); j.richter85@hotmail.com (J.R.); cdoering@physik.uni-kl.de (C.D.)

**Keywords:** III/V semiconductors, reflectance anisotropy spectroscopy (RAS), reactive ion etching (RIE), etch-depth monitoring, etch-stop indicator layers, quantum dots

## Abstract

Reflectance anisotropy spectroscopy (RAS), which was originally invented to monitor epitaxial growth, can—as we have previously shown—also be used to monitor the reactive ion etching of III/V semiconductor samples in situ and in real time, as long as the etching rate is not too high and the abrasion at the etch front is not totally chaotic. Moreover, we have proven that—using RAS equipment and optical Fabry‒Perot oscillations due to the ever-shrinking thickness of the uppermost etched layer—the in situ etch-depth resolution can be as good as ±0.8 nm, employing a Vernier-scale type measurement and evaluation procedure. Nominally, this amounts to ±1.3 lattice constants in our exemplary material system, AlGaAsSb, on a GaAs or GaSb substrate. In this contribution, we show that resolutions of about ±5.6 nm can be reliably achieved without a Vernier scale protocol by employing thin doped layers or sharp interfaces between differently doped layers or quantum-dot (QD) layers as etch-stop indicators. These indicator layers can either be added to the device layer design on purpose or be part of it incidentally due to the functionality of the device. For typical etch rates in the range of 0.7 to 1.3 nm/s (that is, about 40 to 80 nm/min), the RAS spectrum will show a distinct change even for very thin indicator layers, which allows for the precise termination of the etch run.

## 1. Introduction

### 1.1. General Remarks

Reactive ion etching (RIE) is a core technological process of lithographic semiconductor device fabrication, increasing accuracy and reproducibility in comparison to wet etching [1,2,3]. Typically, a small amount of an appropriate reactive gas (for example, Cl_2_ or CF_4_) is mixed with a larger portion of a nonreactive, so-called plasma gas (usually argon) inside a vacuum chamber.

Most electronic as well as micromechanical devices are based on silicon. Optoelectronic devices, such as semiconductor lasers, often rely on sophisticated sequences of monocrystalline cubic III/V semiconductor layers with heterostructures, intrinsic (i) and doped (n, p) layers, quantum films, and quantum dots (QD) [4,5,6]. Depending on the complexity and functionality of the device under consideration, the dry-etch process might have to be terminated at a certain depth with accuracies better than tens of nanometers [5]. Sometimes, the etching process should even be stopped at an interface between two layers, for example, layers of the same material composition, but with different doping levels and/or different dopants, that is, p-GaAs/i-GaAs or p-GaAs/n-GaAs. The task will get even harder if the accuracy is to be achieved in situ and in real time in order to increase the process yield.

Reflectance anisotropy spectroscopy (RAS) is a nondestructive optical and surface-sensitive technique well established for monitoring epitaxial and dry-etch processes [5,6,7,8,9]. For example, the use of RAS equipment has had enormous success in terms of monitoring epitaxial growth, providing information about thickness, doping, the presence of quantum dots, and surface re- or deconstruction [5,6,10,11,12].

The RAS measurement principle involves the use of broad-band linearly polarized light impinging onto the sample surface normally. In principle, normal incidence should not allow for the definition of a plane of incidence and of corresponding directions of linear polarization (parallel or perpendicular to the plane of incidence). However, anisotropies on the surface (that is, on the growth or etch front) break the symmetry [13]. They not only allow for the distinction of different directions of linear polarization, but also alter the state of polarization slightly upon the reflection of the light [6]. Thus, the reflected light will no longer be linearly polarized, but rather reflected slightly elliptically. The difference in reflected intensities for the light field components along two main crystal axes, for example, (110) and (−110) can be used to measure the ellipticity/eccentricity.

Equation (1) calculates the “genuine RAS signal” for a particular photon energy *hν*, where the quantities *R* represent the appropriate reflectivities/reflectances and *x* and *y* stand for the directions of the main crystal axes:

(1) $$\frac{∆R}{\langle R\rangle }(h\nu )=\frac{R_{x}{-R}_{y}}{{(R}_{x}{+R}_{y})/2}.$$

Actually, the RAS signal could in principle even be defined with the amplitude reflection coefficients *r* instead of the reflectivities *R = |r|^2^*. However, phase distortions due to the mechanical tension of the viewport windows of the vacuum chambers deteriorate the measurements, so that most RAS users follow Equation (1).

In [5], the authors report the practical use of RAS *equipment* for high-resolution, in situ, real-time etch-depth monitoring. When employing optical Fabry-Perot oscillations due to the ever-shrinking thickness of the currently etched layer, the achieved etch depth resolution was around ±16 nm. In [14], we have described an improvement of the resolution using Fabry‒Perot oscillations in combination with a Vernier-scale-type evaluation procedure. In the last stage, we used five different photon energies, which give Fabry‒Perot oscillations with five slightly different oscillation periods (or one scale plus four different Vernier scales simultaneously) and allowed for an extremely good resolution of ±0.8 nm.

In the case of III/V semiconductors RIE-RAS (in situ and in real time) monitors the erosion of the surface and can easily detect the interfaces between adjacent layers, for example, due to electric dipoles on the surface or strain. The latter might even stem from a layer just a few nanometers thick [13,15,16,17].

As we will show here, p- and n-doping of the III/V semiconductors lead to a significant unambiguous contribution to the RAS spectra, similar to the case for RAS upon epitaxial growth [16,18,19,20]. Likely, this is due to a piezoelectric contribution via asymmetric distortion of the bonds in the space charge region [16]. The doping type as well as the doping level can be distinguished. In this way, the achieved resolution is extremely good and on the order of ±5.6 nm, even without using a Vernier scale. The fingerprints of these signals can be observed either in the transients of the RAS signal for specific photon energies or in the temporal evolution of the color-coded RAS spectrum, that is, in the so-called color plot.

### 1.2. RAS Color Plot, Spectra, and Transients

**Figure 1:**
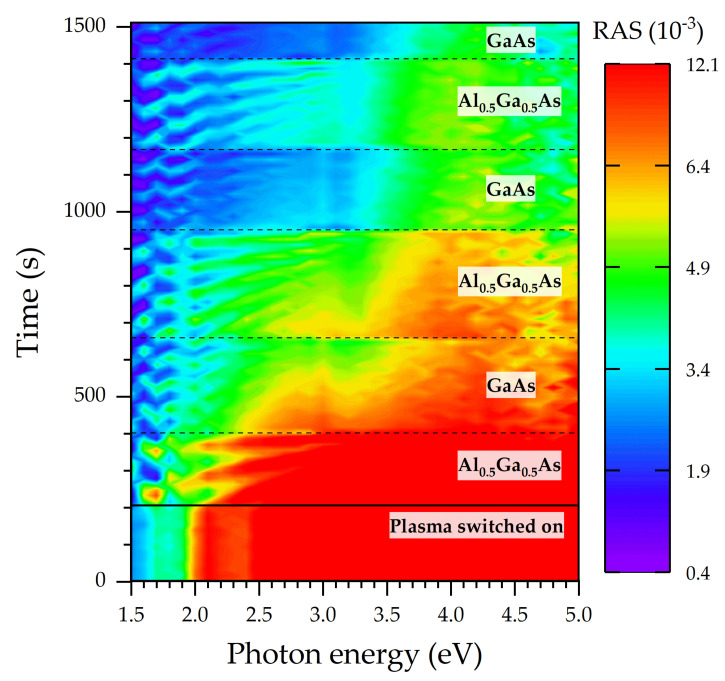
RAS color plot acquired during an RIE process for a sequence of Al_0.5_Ga_0.5_As layers interspersed with GaAs on a GaAs substrate. Black dashed lines mark interfaces between layers.

Figure 1 shows the Fabry-Perot oscillations in a genuine RAS signal from an Al_0.5_Ga_0.5_As/GaAs multilayered sample on the GaAs substrate etched by RIE. The abscissa and the ordinate axis are the photon energy and (etch) time, respectively. Signal heights are color-coded, yielding the RAS color plot.

**Figure 2:**
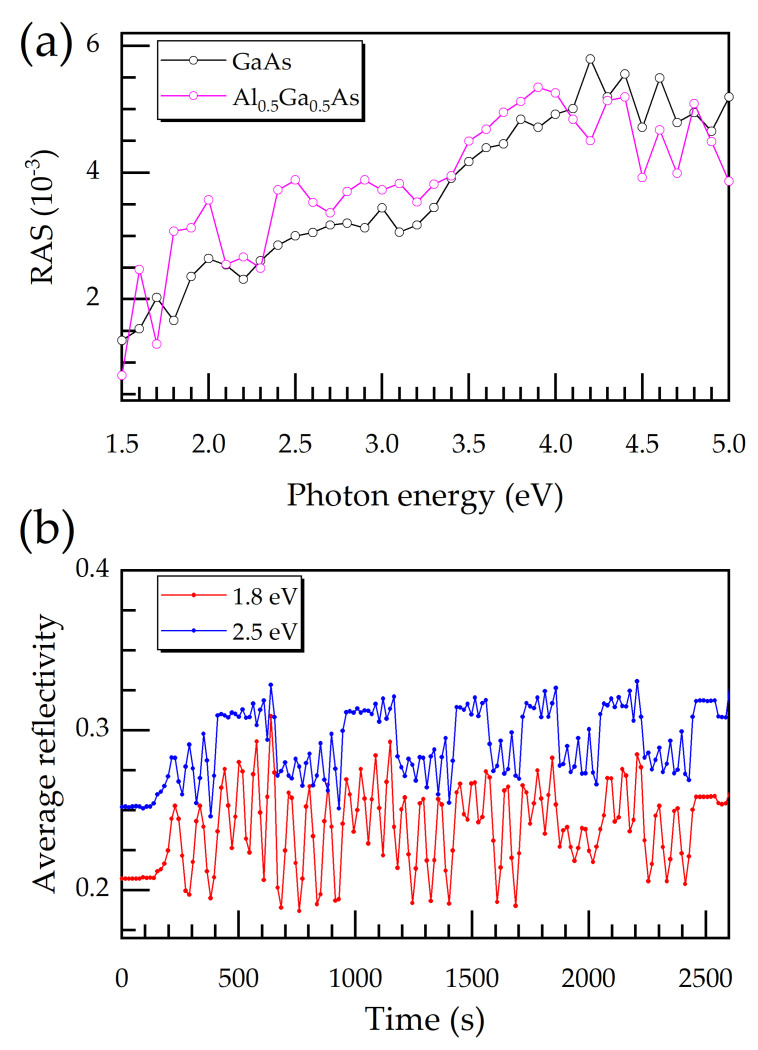
(**a**) RAS spectra for *t_1_* = 1007 s and *t_2_* = 1243 s, taken from Figure 1, which correspond to GaAs and Al_0.5_Ga_0.5_As, respectively. (The lines between experimental data points are just guides for the eye.) (**b**) Transients of the average reflectivity (the denominator in Equation (1)) of the GaAs/Al_0.5_Ga_0.5_As multilayered sample acquired with two photon energies, 1.8 and 2.0 eV.

For the sake of comparison, Figure 2a shows two representative RAS spectra for times *t_1_* = 1007 s and *t_2_* = 1243 s, taken from Figure 1, corresponding to the etching of a GaAs and an Al_0.5_Ga_0.5_As layer, respectively. The spectra for GaAs reveal typical profiles [21]. The general tendency is that signal height increases with photon energy. Deviations from this trend (if not attributable to Fabry-Perot oscillations) must be interpreted as the RAS fingerprint of the material composition [16]. The difference between a RAS spectrum for GaAs and Al_0.5_Ga_0.5_As is due to the substitution of Al atoms for Ga atoms in the lattice, which alters the electric dipole moment, changing the optical reflectivity.

Figure 2b illustrates the RIE-RAS transients of the average reflectivity (the denominator in Equation (1)) of the multilayered GaAs/Al_0.5_Ga_0.5_As sample for two photon energies. The oscillatory behavior over time clearly reveals the Fabry–Perot oscillations due to the etching of the uppermost layer and, hence, its ever-shrinking thickness [5]. It is observable in Figure 2b that the Fabry-Perot oscillation for each etched layer has an average signal value. The mean value of the signal jumps from one level to another, indicating a change in the material composition or doping.

Of course, the oscillation period depends on the etch rate, which is typically in the range of 0.7–1.3 nm/s (40–80 nm/min) for our III/V etch processes. It is important to mention that—once the impinging light penetrates into the semiconductor material—the wavelength changes from its vacuum value *λ* to its material value, *λ_n_ = λ/n(λ)*, with *n(λ)* as the wavelength dependent refractive index. The oscillatory transients constitute a very effective way to monitor the etch process and etch depth in situ and in real time. The changes in the spectrum (for the genuine RAS signal or the average reflectivity) allow to assess the layer composition and appearance of interfaces, and, as we will show here, doping and quantum dots (QD). Thus, interfaces between layers with different composition or doping as well as QD layers can act as etch-stop indicators. For this purpose, layers can be used, which are part of the device design anyway, such as pn-junctions. Alternatively, additional layers can be introduced in the design and during epitaxial growth for this purpose.

### 1.3. Statistical Data Analysis

For this contribution, the promptness of the temporal changes of the “genuine RAS signal” transient or a transient of the average reflectivity will be of great importance. It determines the possible temporal resolution in recognizing the etch-stop indicator and stopping the etch process. We approximate the temporal change from one level of the signal *s* to another by a function resembling the Fermi distribution:

(2) $$s\left(t\right)=\frac{1}{1+e^{\frac{t-t_{0}}{\tau }}}\;\mathrm{or}\;s\left(t\right)=\frac{1}{1+e^{\frac{t_{0}-t}{\tau }}}\;,$$

where *t_0_* stands for the inflection point at the interface to the next layer (or comparable) and *τ* is the time constant for normalization. The time of the inflection point also indicates when the signal has changed by 50% of the difference between both signal levels. The above mentioned two versions of the equation must be considered because the signal upon etching can jump from a higher to a lower level or vice versa. The time constant *τ* might also be interpreted as the temporal resolution for our purposes and, when multiplied by the etch rate, as the etch depth resolution.

Equation (3) represent the first temporal derivative of Equation (2):

(3) $$\frac{ds}{dt}\left(t\right)=-\frac{1}{2\tau \left[1+\cosh\left(\frac{t-t_{0}}{\tau }\right)\right]}\;\mathrm{or}\;\frac{ds}{dt}\left(t\right)=+\frac{1}{2\tau \left[1+\cosh\left(\frac{t-t_{0}}{\tau }\right)\right]}\;.$$

The derivatives are symmetric curves (bell-shaped or with the shape of an inverse bell) and also allow for the determination of *t_0_* and *τ*.

## 2. Materials and Preparation of Setup

All the samples used for this contribution were grown by molecular beam epitaxy (MBE) in an R450 MBE system from DCA Instruments Oy (Turku, Finland). Two-inch (100) GaAs substrates were overgrown with a GaAs buffer. Then, a sequence of layers was grown with a combination of intrinsic or doped layers as part of complex heterostructures or with GaSb quantum dot (QD) layers. In some cases, the layers were n-doped with tellurium (Te) or p-doped with beryllium (Be), both at doping concentrations in the range of 10^17^ to 10^19^ cm^−3^. The GaSb QD were grown by MBE with nominal coverage between 1 ML (monolayer) and 6 ML, embedded in GaAs layers. The epitaxial growth was monitored in situ and in real time by a commercial RAS system, that is, the EpiRAS TT (Laytec, Berlin, Germany), with a photon energy range of 1.5 to 4.5 eV with a step size of 0.3 eV. A complete spectrum can be measured within 45 s. Of course, in the case of transients, not all photon energies have to be used and the data acquisition gets faster.

After epitaxy, the samples were diced/cleaved into several pieces of about 8 mm^2^. The reactive ion dry-etch process was carried out in a MicroSys 350 parallel plate reactor (Roth & Rau, Wuestenbrand, Germany) using a bias voltage of 500 V. The plasma contained a gas mixture of 98% Ar and 2% Cl_2_, adjusted using a mass flow controller maintained at 13 sccm, whereas the pressure varied in the range of 8.9·10^−3^ to 1.2·10^−2^ hPa [22]. For RAS control of the etch processes, another Laytec RAS system was used, i.e., an EpiRAS 200 (Laytec). However, the range of photon energies used extended from 1.5 to 5.0 eV with a minimum step size of 0.05 eV. The RAS system was positioned above a viewport perpendicular above the sample in the RIE machine. Before each etch process, the RAS system was adjusted to the optimum angle of 45° of the direction of linear light polarization with respect to the main crystal axes in order to maximize the signal height. For this purpose, the RAS system was rotated around its optical axis and fixed to its optimal orientation once before etching.

## 3. Results and Discussion

Section 3.1 gives RAS results during epitaxial growth of GaAs samples with n- and p-doping or with GaSb QD layers. Section 3.2 deals with RAS results upon reactive ion etching of a semiconductor laser sample, which contains layers with different doping levels interspersed with GaSb QD layers. In Section 3.3 and Section 3.4, the applicability of the method with etch-stop indicators is discussed, also giving values for the temporal resolution, which can be translated into a resolution of the etch depth. This latter resolution gives the etch depth uncertainty upon etch-stop.

### 3.1. RAS Signal Fingerprints of Doped GaAs Layers and GaSb QD upon Epitaxial Growth

In the case of GaAs as host material, doping impurities can replace Ga or As atoms in their respective sites and thus alter the mechanical strain and hence the electric dipole moments at the surface. In the case of pn-junctions, even the space charge regions might change the picture [16].

**Figure 3:**
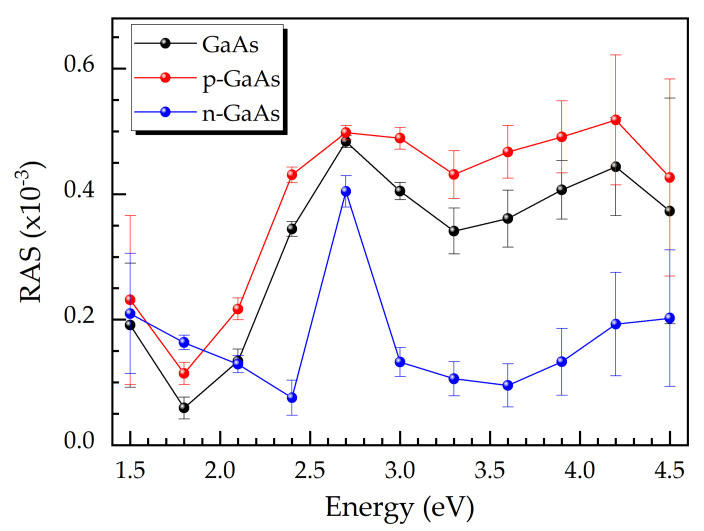
RAS spectra of intrinsic, p-, and n-doped GaAs acquired during the epitaxial growth. The experimental data are black, blue, and red dots, while the straight lines are just guides for the eye.

Figure 3 shows RAS spectra of a p-GaAs layer, an n-GaAs layer, and an intrinsic GaAs sample acquired during epitaxial growth. The doping concentration of the p-GaAs layer is slightly above 10^19^ cm^−3^, whereas that for the n-GaAs layer is about 7·10^18^ cm^−3^. The RAS spectrum for intrinsic GaAs exhibits the typical signature due to the anisotropies on the semiconductor surface [23,24,25]. The RAS spectrum of the p-GaAs layer has a higher RAS signal for all photon energies. In the case of the n-GaAs layer, there is a distinct peak at about 2.4 eV in the spectrum. Taking photon energies above 2 eV allows for a distinction between the p- and the n-type material.

**Figure 4:**
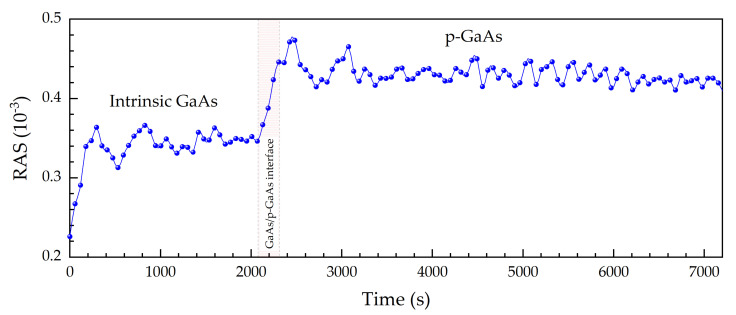
RAS signal at 2.4 eV as a function of time upon epitaxial growth. The red shaded region indicates the growth of the interface between the intrinsic GaAs and the p-GaAs layer.

Figure 4 illustrates the temporal evolution of the RAS signal at 2.4 eV, again during epitaxial growth. The RAS signal for the intrinsic GaAs layer has a plateau at around 0.34·10^−3^. The intensity rises after 2075 s, that is, when Be atoms are embedded in the crystal lattice. At this point, the impurities increase the anisotropy at the surface by provoking an asymmetric distortion of the chemical bonds [16]. The slow GaAs/p-GaAs interface growth took approximately 227 s, which corresponds to a growth thickness of about 20 nm. The transition time is mainly due to the growth process itself, not the RAS response.

**Figure 5:**
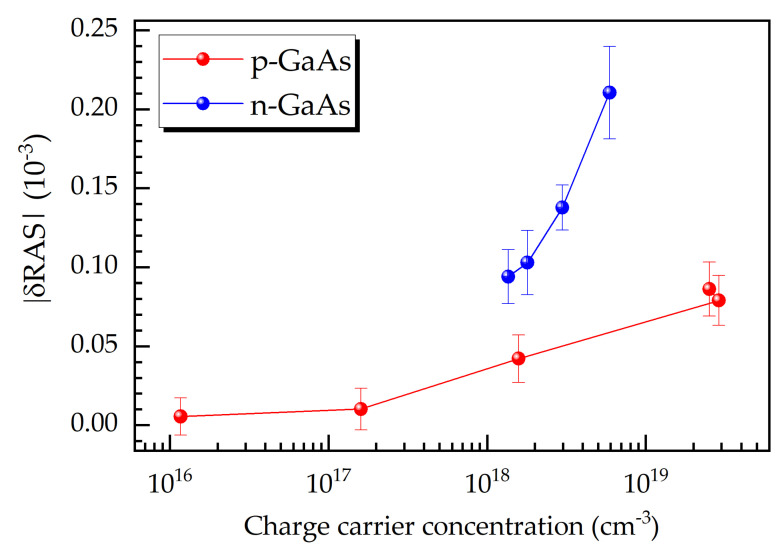
Correlation between charge carrier concentration and the absolute value of the RAS signal difference, |δRAS|, at 2.4 eV for p- and n-GaAs.

The sensitivity of RAS with respect to doping impurities allows for the determination of doping concentrations (after calibration runs). The semilogarithmic graph in Figure 5 reveals the strong dependence of the quantity |δRAS| at 2.4 eV on the doping or majority charge carrier concentration, where |δRAS| is the difference between the RAS signal of the doped and the intrinsic GaAs layers in absolute values. The results also show that the quantity |δRAS| is more sensitive to the doping level for the n-type material than for the p-type one.

**Figure 6:**
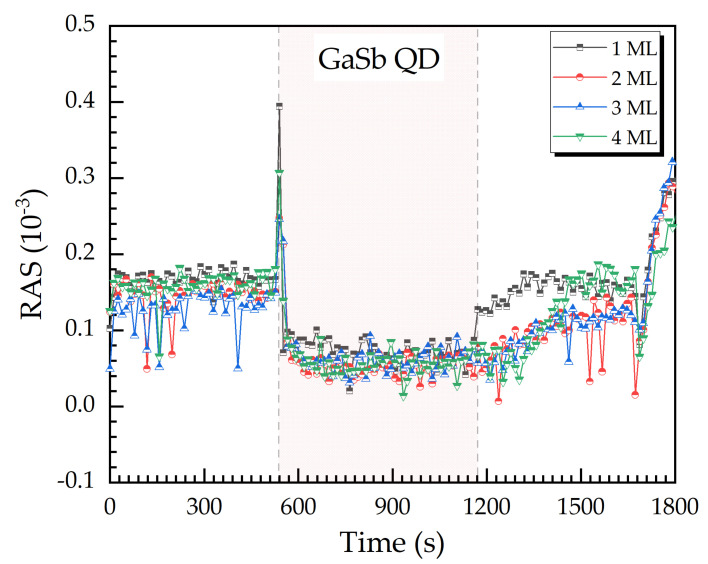
RAS transients at 2.7 eV acquired during the epitaxial growth process of GaSb QD layers, varying the nominal coverage between one and four monolayers (ML).

In Figure 6 four RAS signal transients at 2.7 eV are given for the Stranski-Krastanov growth of Ga(As)Sb quantum dot (QD) layers with different nominal coverages from 1 to 4 ML (monolayer). The red shaded region represents the duration of the QD growth. The nominal coverage gives the amount of material offered to the surface for QD growth. In our material system, QD growth might start with the evolvement of a so-called wetting layer of the QD material, before the QD themselves emerge. Typically, we find wetting layers of 0–1 ML [4].

In all four cases, the RAS signal shows a significant peak, as soon as the wetting layer or the QD layer starts to grow. The difference in the lattice constant is large enough to induce strain detectable by RAS [19,24]. The subsequent increase of the RAS signal slightly later, after the low plateau, can be interpreted as being related to crystal lattice relaxation.

### 3.2. In Situ RAS Monitoring during RIE of a III/V Semiconductor Laser

For comparison with the RAS growth results from Section 3.1, we etched a complete layer sequence of a semiconductor laser with pn-junction and QD layers as active material. The layer sequence from top to bottom (in the order relevant for etching) was: a lightly p-doped Al_0.5_Ga_0.5_As cap, p-Al_0.5_Ga_0.5_As, eight layers of GaSb QD (as active material) embedded in 50-nm-thick GaAs barrier layers, n-Al_0.5_Ga_0.5_As, and a lightly n-doped Al_0.5_Ga_0.5_As buffer, as well as the (100) GaAs substrate. The doping carrier concentrations of the heavily doped p-Al_0.5_Ga_0.5_As and n-Al_0.5_Ga_0.5_As layers were around 10^17^ and 10^18^ cm^−3^, respectively. The nominal coverage for the GaSb QD growth was 2 ML for all QD layers.

**Figure 7:**
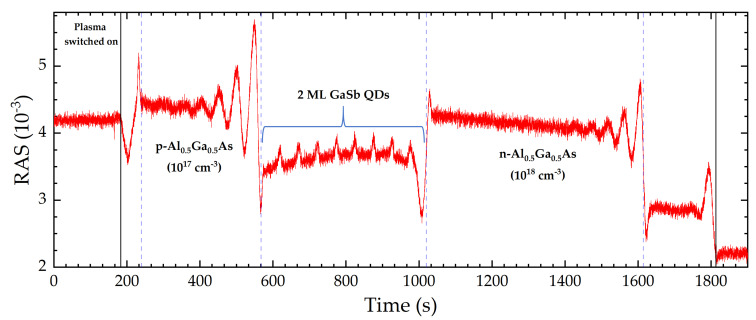
RAS signal at 3.05 eV from semiconductor laser sequence upon reactive ion etching. The eight 2 ML GaSb QD layers can be clearly detected in the structure.

For RAS monitoring, a photon energy of 3.05 eV was used, because this value allows for the detection of both the doping and the QD layers. Figure 7 illustrates the RAS signal transient upon etching. The solid black vertical lines mark the points in time when the plasma has been switched on and when the buffer layer has been removed completely. The mean etch rate is 1 nm/s (60 nm/min), as calculated from Fabry-Perot oscillations.

The presence of GaSb QD layers was revealed by several peaks of the RAS signal, each followed by an abrupt drop. This behavior reminds us of the signal transient from the growth, but in reversed order—as it should be for etching. As soon as any QD layer is completely etched away, the signal goes back to its background value. The ascents of the RAS intensity from the baseline to the maximum values take about 10 s each, which corresponds to a QD height/depth of about 10 nm.

It is important to stress that, for Figure 7, a realistic laser layer sequence has been etched with standard reactive ion etching parameters. This result sheds light on the possibility of using a change in doping as well as the occurrence of QD layers as etch-stop indicators. The resolution of this method is discussed in Section 3.3 and Section 3.4

### 3.3. GaSb QD Layer as an Etch-Stop Indicator

**Figure 8:**
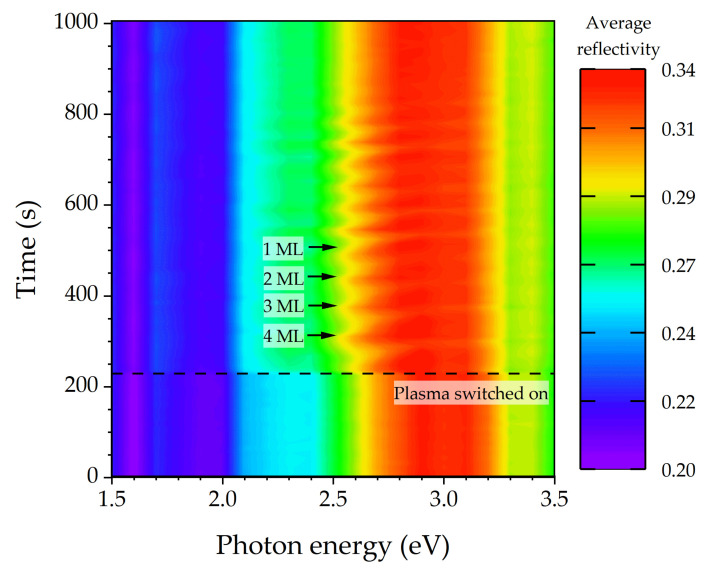
Color plot of average reflectivity during the reactive ion etching of a sample with three stacks of GaSb QD layers with 4, 3, 2, and 1 ML nominal coverage embedded in a 50-nm-thick GaAs barrier and leveling layers. The black arrows indicate the time when the fluctuations in the optical signal from the first stack of GaSb QD layers are observed. The most prominent features appear at 2.6 eV photon energy.

Figure 8 contains the color plot from the etch process, using a sample with three-layer stacks, each made of four GaSb QD layers with 4, 3, 2, and 1 ML nominal coverage (order upon etching) embedded in 50-nm-thick GaAs layers. The appearance of the QD fingerprints for the first stack (from the top) is marked by black arrows. In this case, the etch rate can be estimated from the etch time for the GaAs layers and amounts to 0.7 nm/s (around 40 nm/min). The acquisition time of any RAS spectrum is around 10 s for this measurement. That means that, during the acquisition of a single spectrum, the material is etched/eroded by about 7 nm.

The strong signal changes at about 2.6 eV photon energy are not necessarily directly related to the etching of the QD layers, as they might be related to a Fabry-Perot oscillation. This has to be checked first. The vacuum wavelength corresponding to 2.6 eV is *λ* = 477 nm. With a refractive index of *n* = 4.49 for GaAs and this wavelength, the wavelength inside the material is *λ_n_* = 106 nm. Since the light is reflected, we would expect a Fabry-Perot oscillation period corresponding to any additional etch depth of *λ_n_*/2 ≈ 50 nm, which happens to be the thickness of the intermediate and leveling GaAs barrier layers. Thus, there might be ambiguity about whether the signal oscillation is due to the periodic QD layer/barrier structure or the shrinking Fabry-Perot resonator thickness. However, the change of the Fabry-Perot oscillation period beyond 2.6 eV is a strong argument that the signal oscillations are indeed Fabry-Perot oscillations. The signal at 2.6 eV should be observed in more detail.

**Figure 9:**
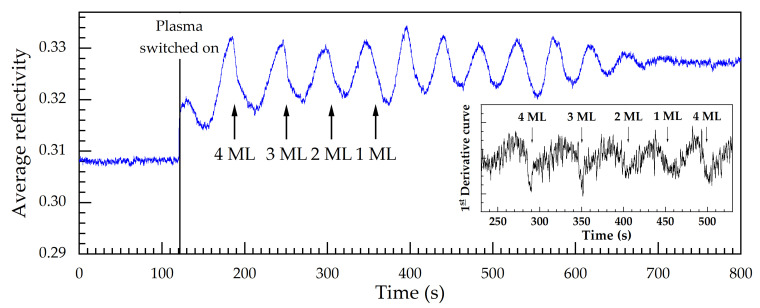
Average reflectivity as a function of time at 2.6 eV photon energy for the sample with three stacks of GaSb QD layers with 4 ML, 3 ML, 2 ML, and 1 ML nominal coverage embedded in 50-nm-thick GaAs barrier and leveling layers. The inset shows the first time derivative of the average reflectivity, where discontinuities are clearly observed due to QD layers.

Thus, Figure 9 is used to show a transient of the average reflectivity at 2.6 eV photon energy, where the features are most prominent in the color plot of Figure 8. The dashed vertical line indicates the plasma switch-on. The transient has been achieved during another etch process (beyond that for Figure 8), when only the data for this photon energy have been collected. As mentioned before, to use a transient instead of a color plot upon etching increases the temporal resolution, which plays a central role in pushing up the resolution limit (in etch time or etch depth).

As expected from growth, the average reflectivity for etching—in addition and as a modification to the layer stack or Fabry-Perot oscillation—exhibits sharp descents for the QD layers with 4 and 3 ML nominal coverage, see the leftmost two black arrows in Figure 9. For the case with 1 ML, a sharp descent is not observed; this might be connected to the possibility that 1 ML coverage might not yet lead to QD growth, but rather to a strained wetting layer. For the case with 2 ML nominal coverage, one might argue about the occurrence of a steep descent. In the inset of the figure, the time derivative of the average reflectivity is shown for part of the etch process in order to make the signal changes more prominent. Again, the influence of the QD layers is prominent for 3 and 4 ML nominal coverage, can be argued upon for 2 ML, and is not seen for 1 ML. We conclude that QD layers can be employed as RAS etch-stop indicators for nominal coverages above 2 ML in our material system.

**Figure 10:**
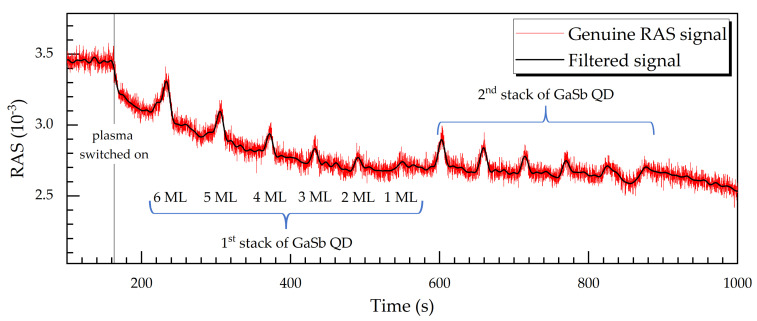
Genuine RAS signal at 3.05 eV photon energy as a function of time for a sample with two stacks of GaSb QD layers with 6, 5, 4, 3, 2, and 1 ML nominal coverage each, embedded in a 50-nm-thick GaAs barrier and leveling layers. The etch rate is around 0.7 nm/s (40 nm/min), leading to a resolution of around 16.3 nm as an etch-stop indicator. The RAS signal has been low-pass-filtered using a fast Fourier transform algorithm with a cutoff frequency of 84 mHz.

In Figure 8 and Figure 9, we have been dealing with the average reflectivity (the denominator in Equation (1)). Now we are going to have a closer look at the genuine RAS signal again. Figure 10 shows a RAS signal transient (red curve) at 3.05 eV from the RIE process of another sample, this one with two stacks of GaSb QD layers with 6, 5, 4, 3, 2, and 1 ML nominal coverage, again embedded in a 50-nm-thick GaAs barrier and leveling layers. Moreover, the RAS signal is low-pass-filtered using an FFT analysis. There are 12 peaks in the transient, which correspond to two times six GaSb QD layers. The intensity of the signal is proportional to the nominal coverage; that is, the highest peaks correspond to the two cases of 6 ML nominal coverage.

In this case, we do not observe Fabry-Perot oscillations because we purposely used a photon energy (3.05 eV), where Fabry-Perot oscillations are not prominent. It is remarkable that, even with the genuine RAS signal, the occurrence of thin GaSb QD layers with 2 or even 1 ML coverage can be detected. This makes the use of QD layers as etch-stop indicators even more appealing. Thus, the resolution limit should be on the order of the growth time for a QD layer of even just 1 ML, which amounts to 13.6 s, corresponding to an etch-depth of 16.3 nm.

### 3.4. Resolution Limit of III/V Doped Layers as Etch-Stop Barrier Indicators upon RAS-RIE

**Figure 11:**
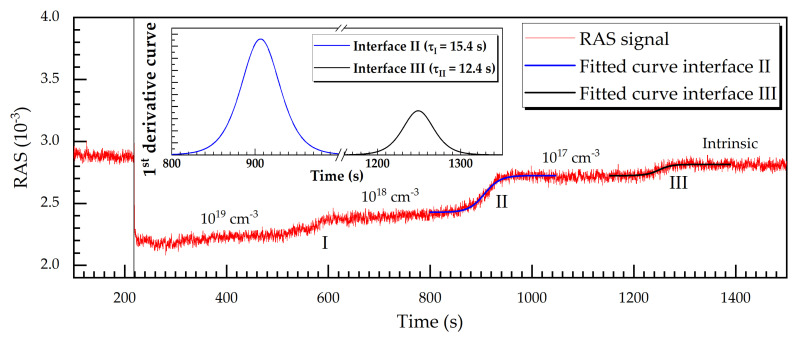
RAS transient acquired at 3.05 eV photon energy during the RIE process for an n-GaAs layer with a charge carrier concentration between 10^17^ to 10^19^ cm^−3^. The RAS signal along interfaces II and III is fitted using Equation (2), and their first derivatives according to Equation (3) as shown in the inset.

The RAS signal magnitude changes according to the doping type and level. Figure 11 illustrates a RAS signal transient at 3.05 eV, red curve, for an n-doped GaAs layer, etched with a rate of 1.2 nm/s (about 70 nm/min). The plasma ignition point in time is marked by the black solid vertical line after about 220 s into the process.

The temporal evolution of the RAS signal exhibits four plateaus, which correspond to the doping carrier concentrations of about 10^19^, 10^18^, and 10^17^ cm^−3^, and to intrinsic GaAs. Since the semiconductor layers are made of the same material (that is, GaAs), the average reflectivity is not appropriate to detect variations in the doping level. It is more appropriate to use the genuine RAS signal itself. The effect of the doping and the different doping levels can clearly be observed in Figure 11.

For interfaces II and III, Equation (2) from Section 1.3 are applied and its time derivatives according to Equation (3) are represented by the blue line (II) and the black line (III) in the inset of Figure 11. The quantity *τ* from Equations (2) and (3) can be used as a figure of merit for the temporal etch resolution (etch time). According to this definition, the etching of interface II had a time constant of *τ* = 15.4 s, corresponding to a ±18.5 nm etch depth (considering the etch rate of 1.2 nm/s (72 nm/min)) and an equivalent etch depth resolution. Similarly, for interface III we deduced an etch depth resolution of ±14.8 nm. This is a very good resolution for an etch-stop barrier, considering in situ and real-time monitoring.

The broad interface width is related to the extent of the space charge regions. However, if the signal change is only intended to indicate a change in doping, a complete transition does not have to be awaited. Thus, the above mentioned resolutions are an upper limit.

**Figure 12:**
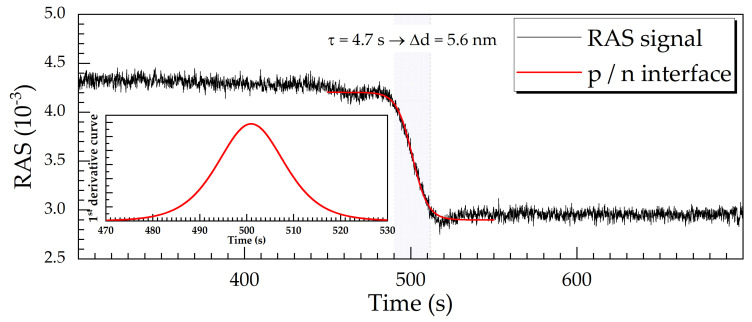
An RAS signal transient at 3.05 eV upon an RIE process for a pn-junction (diode). The red curve has been fitted using Equation (2); see also the inset for the time derivative according to Equation (3).

In electronic and optoelectronic devices, pn-junctions are common subunits. Thus, we describe another result here, which is related to pn-junctions. Figure 12 shows the temporal evolution of the RAS signal at 3.05 eV during the etching across a GaAs pn-junction. The charge carrier concentration is 10^18^ cm^−3^ for the p-type and 10^17^ cm^−3^ for the n-type material, which results in an RAS signal difference of about 1.3·10^−3^ between the top and the bottom plateau. The RAS signal has a perfect shape according to Equation (2). Following the same criteria adopted above, the time constant gives an etch depth resolution of ±5.6 nm. This is an extremely good result for in situ monitoring of the etching up to (and of course beyond) a pn-junction, even when compared to ex situ techniques.

## 4. Conclusions

Reflectance anisotropy spectroscopy (RAS) is a powerful surface-sensitive tool to monitor, in situ and in real time, the epitaxial growth (here MBE) as well as reactive ion etching (RIE) of crystalline III/V semiconductor samples. As an example, we have used the AlGaAs(Sb) material system on GaAs substrates.

For epitaxy, doping types become distinguishable by employing RAS photon energies from 2.0 to 4.5 eV. It is also possible to estimate the doping level according to the magnitude of the mean RAS signal after previous calibrations [20]. It is also possible to use the RAS signal for identification of quantum dot (QD) layer growth for nominal coverages of more than 1 or 2 ML.

RAS can also be employed to detect the doping type, doping level, and occurrence of a QD layer upon reactive ion etching (RIE). Thus, appropriate interfaces and QD layers might be used as etch-stop indicators. If not part of the layer sequence design, they might be added to the layer design just for this purpose.

Depending on the exact conditions, the etch depth and thus the etch-stop resolution might be between ±14.8 nm or even ±5.6 nm, which is sufficient for most applications in semiconductor technology.

## Data Availability

The data that support the findings of this study are available on request from the corresponding author H.F.
